# Differentiating tracheobronchial involvement in granulomatosis with polyangiitis and relapsing polychondritis on chest CT: a cohort study

**DOI:** 10.1186/s13075-022-02935-2

**Published:** 2022-10-28

**Authors:** Carole Jalaber, Xavier Puéchal, Ines Saab, Emma Canniff, Benjamin Terrier, Luc Mouthon, Eglantine Cabanne, Sandra Mghaieth, Marie-Pierre Revel, Guillaume Chassagnon

**Affiliations:** 1 Radiology Department, Hôpital Cochin, AP-HP, 27 rue du Faubourg Saint-Jacques, 75014 Paris, France; 2 Internal Medicine Department, Referral Center for Rare Systemic Autoimmune Diseases of Ile de France, Hôpital Cochin, AP-HP, 27 rue du Faubourg Saint-Jacques, 75014 Paris, France; 3 Université de Paris, 27 rue du Faubourg Saint-Jacques, 85 boulevard Saint-Germain, 75006 Paris, France

**Keywords:** Multidetector computed tomography, Relapsing polychondritis, Granulomatosis with polyangiitis, Trachea, Respiratory tract diseases

## Abstract

**Background:**

In patients with tracheobronchial involvement, the differential diagnosis between granulomatosis with polyangiitis (GPA) and relapsing polychondritis (RP) can be challenging. The aim of this study was to describe the characteristics of airway abnormalities on chest computed tomography (CT) in patients with GPA or RP and to determine whether specific imaging criteria could be used to differentiate them.

**Methods:**

GPA and RP patients with tracheobronchial involvement referred to a national referral center from 2008 to 2020 were evaluated. Their chest CT images were reviewed by two radiologists who were blinded to the final diagnosis in order to analyze the characteristics of airway involvement. The association between imaging features and a diagnosis of GPA rather than RP was analyzed using a generalized linear regression model.

**Results:**

Chest CTs from 26 GPA and 19 RP patients were analyzed. Involvement of the subglottic trachea (odds ratio for GPA=28.56 [95% CI: 3.17; 847.63]; *P=0.001*) and extensive airway involvement (odds ratio for GPA=0.02 [95% CI: 0.00; 0.43]; *P=0.008*) were the two independent CT features that differentiated GPA from RP in multivariate analysis. Tracheal thickening sparing the posterior membrane was significantly associated to RP (odds ratio for GPA=0.09 [95% CI: 0.02; 0.39]; *P=0.003*) but only in the univariate analysis and suffered from only moderate interobserver agreement (kappa=0.55). Tracheal calcifications were also associated with RP only in the univariate analysis (odds ratio for GPA=0.21 [95% CI: 0.05; 0.78]; *P=0.045*).

**Conclusion:**

The presence of subglottic involvement and diffuse airway involvement are the two most relevant criteria in differentiating between GPA and RP on chest CT. Although generally considered to be a highly suggestive sign of RP, posterior tracheal membrane sparing is a nonspecific and an overly subjective sign.

## Highlights

• The presence of subglottic involvement is in favor of GPA.

• Extensive airway involvement is in favor of RP.

• Posterior tracheal membrane sparing is a nonspecific and an overly subjective sign.

## Background

Granulomatosis with polyangiitis (GPA, formerly known as Wegener’s granulomatosis) and relapsing polychondritis (RP) are two autoimmune diseases that can affect the tracheobronchial tree. GPA is a systemic necrotizing vasculitis, which affects small-to-medium–sized blood vessels, associated with myeloperoxidase (MPO) anti-neutrophil cytoplasm antibodies (ANCA) or proteinase 3 (PR3) ANCA [1]. RP is an immune-mediated systemic disease that affects cartilaginous structures resulting in their progressive destruction, including the ear, nose, respiratory tract, and joint cartilages [2]. Both are rare diseases, with an estimated respective incidence of 0.4 to 11.9 and 3.5 per million inhabitants per year in the USA for GPA [3] and RP [4]. They share several clinical features [5] and exceptional cases of overlap between both disorders can be seen.

Although pulmonary involvement is found in two-thirds of patients with GPA, tracheobronchial involvement is rare, with tracheal stenosis reported in less than 2% of cases [6]. Tracheobronchial involvement is more common in RP where tracheal stenosis is reported in 23% of cases [7]. Computed tomography (CT) is the imaging modality of choice to evaluate the trachea and central airways [8] and it can be used to distinguish subsets of RP based on airway involvement [5].

To date, there are no diagnostic criteria for GPA and RP, but only classification criteria which include pulmonary nodules, mass, or cavitation on chest imaging for GPA [9, 10.] and cartilage inflammation of the ear, nose, or laryngotracheal cartilage for both GPA and RP [7, 9]. In the setting of initial tracheobronchial involvement, the differential diagnosis between the two diseases can be difficult. Most GPA patients have PR3-ANCA, or less frequently MPO-ANCA, which are now regarded as an exclusion criterion for the diagnosis of RP [11]. However, 5–10% of patients have negative results for ANCA immunoassays [3, 6]. In RP, chondritis is the main characteristic abnormality and is required for the diagnosis [11]. However, cartilage involvement may also be seen in GPA [3, 6, 9, 12], thus increasing the complexity in differentiating between the two diseases. This complexity is further compounded by the fact that tracheal stenosis and cartilage involvement of the nose in GPA patients is more frequently observed in localized forms, which are more often negative for PR3-ANCA [13].

In patients with initial tracheobronchial involvement, analysis of tracheobronchial involvement on imaging has been proposed to help differentiate GPA from RP at the early stage. The most commonly reported feature is tracheal wall thickening with sparing of the posterior membrane in RP [14, 15] and subglottic tracheal stenosis in GPA [16, 17]. However, there are no available data on the diagnostic value of imaging criteria to distinguish a diagnosis of GPA from RP. Therefore, the purpose of this study was to describe the characteristics of tracheobronchial involvement in patients with a final diagnosis of GPA or RP and to determine whether specific imaging criteria could be used to differentiate them.

## Materials and methods

### Study design and population

This retrospective observational study was approved by our local ethics committee (CLEP Decision no AAA-2020-08044), which waived the need for patient consent.

All consecutive patients with RP and GPA referred to the National Referral Center for Rare Systemic Autoimmune Diseases at our hospital between February 2009 and July 2020 were included if they had tracheobronchial involvement and an available chest CT. The exclusion criterion was CT images available only in slice thickness of ≥ 5 mm.

### Diagnosis criteria

There are no validated diagnostic criteria for RP. Patients were classified as having RP if they fulfilled two major criteria or one major and two minor criteria according to the Michet classification [7]. Thus, the diagnosis of RP was established on clinical grounds and required either confirmed inflammation in two of three auricular, nasal, or laryngotracheal cartilages or confirmed inflammation in one of the above cartilages and two other minor criteria which include hearing loss, ocular inflammation, vestibular dysfunction, and seronegative polyarthritis. Furthermore, it is now commonly accepted that the exclusion of differential diagnoses, which can mimic RP, is crucial, particularly GPA. Thus, positive ANCA results with PR3 or MPO specificity, involvement of the lung parenchyma, or destructive ear-nose-throat (ENT) lesions in this context additionally classify the patient as having GPA and not RP [11]. For GPA, patients had to meet the 1990 American College of Rheumatology classification criteria requiring evidence of vasculitis and/or revised Chapel Hill Nomenclature [1, 10]. We did not use any airway involvement pattern to differentiate GPA from RP patients.

### CT acquisitions and image analysis

CT images were acquired on several CT scanner models without the standardization of acquisition parameters. All patients had inspiratory volumetric acquisition of at least the entire chest from the lung apices to the diaphragm during a single inspiration. When available, additional expiratory CT images were also analyzed. When several eligible chest CT scans were available, the first scan showing tracheobronchial involvement was chosen for the analysis.

Image analysis was performed independently by two radiologists (CJ and IS) with 2 and 5 years of experience in thoracic imaging, respectively. They reviewed CT images blindly to the final RP or GPA diagnosis, looking for airway wall thickening, tracheobronchial stenosis, tracheobronchial calcifications, tracheobronchomalacia, bronchiectasis, small airway involvement (mosaic attenuation and air trapping), and parenchymal abnormalities, as follows:

- *Airway wall thickness* was visually assessed. In case of thickening, the following features were described: the airway segment involved, the localized or extensive nature (more than 2-cm extension) of the thickening, and the sparing of the posterior tracheal membrane.
- *Airway stenosis* was defined as a narrowing of the lumen diameter of at least 50%. In case of stenosis, the segment involved and its localized or extensive nature were mentioned using the same criteria as for airway wall thickening.
- *Bronchial calcifications* were considered abnormal when they were present within a thickened bronchial wall with an attenuation value of ≥250 Hounsfield units.
- *Tracheobronchomalacia* was assessed only if additional expiratory images were available and defined as narrowing of at least 50% of the lumen diameter in expiration.
- *Bronchiectasis* was defined as a broncho-arterial ratio >1, bronchial visibility within 1cm of the pleural surface, or lack of distal tapering [18].
- *Mosaic perfusion* was defined as a patchwork of regions of differing attenuation [18]. In the setting of GPA and RP, mosaic perfusion was most likely to correspond to small airway disease which was confirmed by the presence of air trapping.
- *Air trapping* was visually defined in patients with additional expiratory CT images available as at least 2 adjacent lobules or at least 5 lobules per lung failing to increase in attenuation at the end of expiration.
- Parenchymal abnormalities included noncalcified centrilobular *pulmonary nodules* and *parenchymal consolidations*.

For lesion location, the tracheobronchial tree was divided as follows: (i) the subglottic trachea (within 10 mm below the glottic plane), (ii) the cervical trachea (below the subglottic level), (iii) the thoracic trachea, (iv) main and lobar bronchi, and (v) distal bronchi (segmental bronchi and beyond). Involvement was considered extensive if it was at least 2 cm long.

In cases of discrepancy between the two observers, a third radiologist with 7 years of experience in thoracic imaging (GC) was responsible for the decision. In addition, based on their experience, the two observers were asked to classify patients as having a CT appearance suggestive of RP, GPA, or indeterminate. All observers were blinded to the initial CT interpretation report and the final diagnosis of RP or GPA.

### Statistical analysis

Statistical analysis was performed using “R” software (version 3.6.3, R Foundation, Vienna, Austria). First, interobserver variability was assessed by Cohen’s kappa coefficient. Additionally, the variability between the two observers and consensus reached between them were assessed. The coefficient was interpreted as follows: values ≤ 0 as indicating no agreement and 0.01–0.20 as none to slight, 0.21–0.40 as fair, 0.41–0.60 as moderate, 0.61–0.80 as substantial, and 0.81–1.00 as almost perfect agreement.

For the rest of the analysis, a CT sign was considered present according to the consensus between observers. The association between imaging features and a diagnosis of GPA rather than RP was studied using a generalized linear regression model. For multivariate analysis, only the variables significantly associated with a diagnosis of GPA with a *p* value <0.05 were included in the model. A *p* value <0.05 was considered significant.

To assess the performance of the observers to differentiate between GPA and RP, CT exams classified as indeterminate were considered misdiagnoses.

## Results

### Study population

**Fig. 1:**
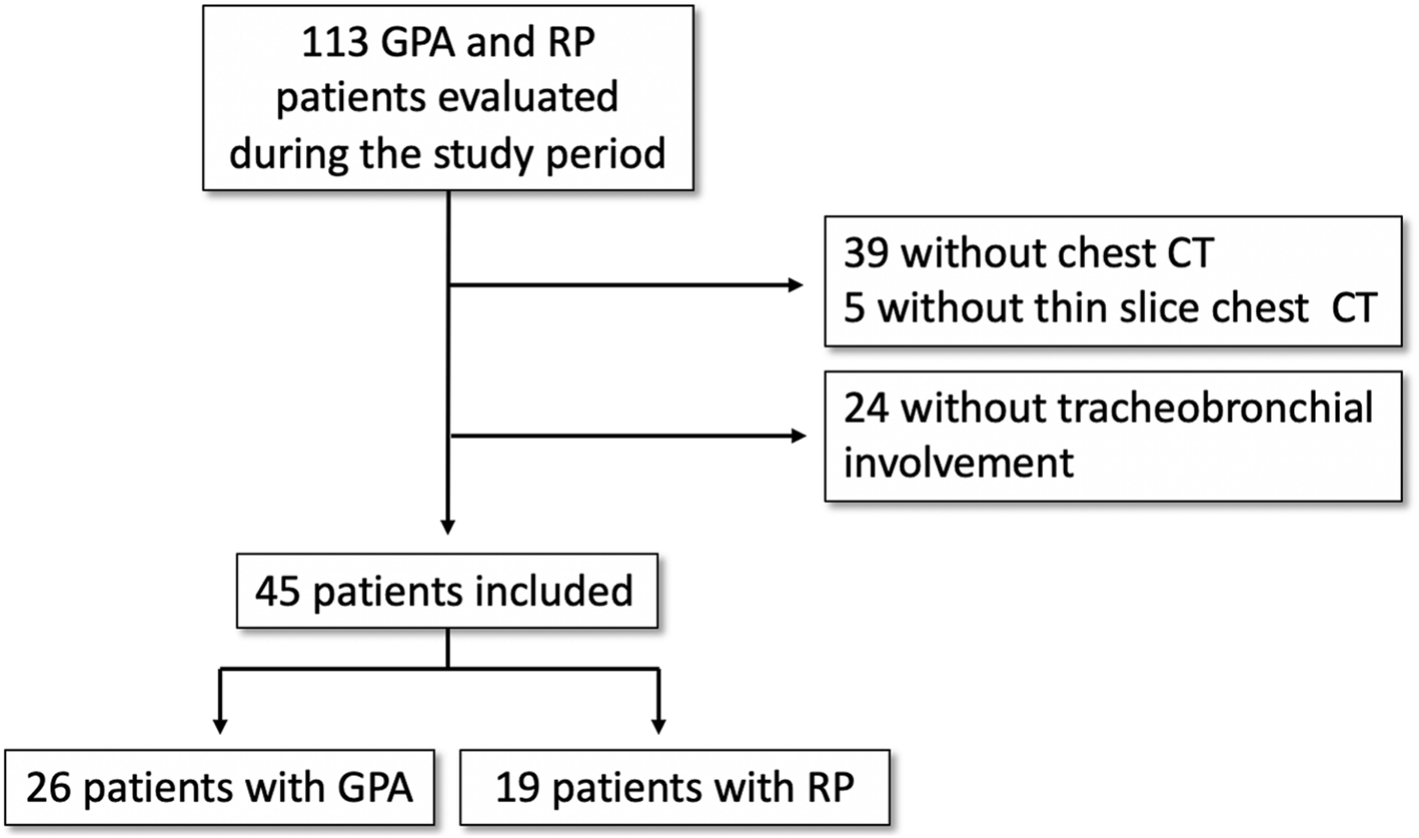
Flow chart (GPA granulomatosis with polyangiitis, RP relapsing polychondritis)

During the study period, 113 patients with RP or GPA were evaluated for suspected tracheobronchial involvement in our referral center for rare systemic autoimmune diseases. Thirty-nine patients were excluded because of the lack of available CT scans. Another 5 patients were excluded because of the absence of thin slice thickness CT images. Of the remaining 69 patients, 45 had tracheobronchial involvement and this constituted our study population (flowchart, Fig. 1). The study population consisted of 26 patients with GPA and 19 with RP. The patients (16 males and 29 females) had a median age of 52 years at the time of the chest CT (interquartile range [IQR]: 36; 64) and had no other autoimmune diseases. Their mean characteristics are shown in Table 1. The majority of the patients with GPA (18/26; 69%) had systemic vasculitis and 8/26 (31%) localized vasculitis. Among GPA patients, 17/25 (68%) were PR3-ANCA positive and 2/25 (8%) were MPO-ANCA positive. Overall, twenty-five out of the 44 patients (60%) with available test results had negative results on immunoassay, namely 24% of the patients with GPA (*n*=6/25) and 100% of the 19 patients with RP.

**Table 1:** Characteristics of the 26 patients with granulomatosis with polyangiitis and 19 with relapsing polychondritis associated with tracheobronchial involvement

	GPA (***n***=26)	RP (***n***=19)
**Age at diagnosis**, *years*	31 [25; 57]	47 [39; 53]
**Age at CT scan**, *years*	42 [31; 63]	53 [47; 66]
**Female**	17/26 (65%)	12/19 (63%)
**Myalgia**	7/26 (27%)	0/19 (0%)
**Arthralgia**	8/26 (31%)	12/19 (63%)
**Fever**	4/26 (15%)	4/19 (21%)
**Weight loss**	8/26 (31%)	2/19 (11%)
**Skin lesions**	4/26 (15%)	4/19 (21%)
**Ocular involvement**	6/26 (23%)	10/19 (53%)
Scleritis	3/26 (12%)	3/19 (16%)
Episcleritis	3/26 (12%)	5/19 (26%)
Uveitis	1/26 (4%)	6/19 (32%)
Others	1/26 (4%)	2/19 (11%)
**Otitis**	11/26 (42%)	2/19 (11%)
**Auricular chondritis**	0/26 (0%)	14/19 (74%)
**Ear deformity**	0/26 (0%)	3/19 (16%)
**Sinusitis**	16/26 (62%)	1/19 (5%)
**Nasal crusts**	19/26 (73%)	2/19 (11%)
**Septal perforation**	2/26 (8%)	0/19 (0%)
**Nasal chondritis**	0/26 (0%)	14/19 (74%)
**Saddle nose deformity**	1/26 (4%)	9/19 (47%)
**Pericarditis**	2/26 (8%)	0/19 (0%)
**Cardiomyopathy**	1/26 (4%)	0/19 (0%)
**Bloody diarrhea**	0/26 (0%)	1/19 (5%)
**Abdominal pain**	0/26 (0%)	1/19 (5%)
**Glomerulonephritis**	5/26 (19%)	0/19 (0%)
**Central nervous system involvement**	1/26 (4%)	0/19 (0%)
**Mononeuritis multiplex**	3/26 (12%)	1/19 (5%)
**Polyneuropathy**	0/26 (0%)	1/19 (5%)
**Autoantibodies**^a^	19/25 (76%)	0/19 (0%)
PR3-ANCA	17/25 (68%)	0/19 (0%)
MPO-ANCA	2/25 (8%)	0/19 (0%)

Qualitative data are presented as proportion with percentage in parentheses, and quantitative data are presented as median with interquartile range in brackets

*GPA* granulomatosis with polyangiitis, *RP* relapsing polychondritis, *ANCA* anti-neutrophil cytoplasm antibodies, *PR3* proteinase 3, *MPO* myeloperoxydase

^a^Immunoassay results were not available for one GPA patient

### Overall imaging findings

The cervical (60%, *n*=27/45) and subglottic trachea (55%, *n*=21/38) were the most frequently involved portions of the tracheobronchial tree, followed by the distal bronchi (51%, *n*=23/45) (Table 2). Tracheal (73%, *n*=33/45) or bronchial (51%, *n*=23/45) thickening was observed in 80% of the patients (*n*=36/45) while stenosis was present in 58% (*n*=26/45). The proportions of tracheal stenosis (31%, *n*=14/45), main or lobar bronchial stenosis (38%, *n*=17/45), and distal bronchi stenosis (33%, *n*=15/45) were of the same range.

**Table 2:** CT findings

	All (***n***=45)	GPA (***n***=26)	RP (***n***=19)	Odds ratio for GPA	***P*** value
**Airway segment involved**					
Subglottic trachea^a^	21/38 (55%)	18/23 (78%)	3/15 (20%)	14.40 [3.22; 85.07]	0.001
Cervical trachea	27/45 (60%)	16/26 (62%)	11/19 (58%)	1.16 [0.34; 3.92]	0.838
Thoracic trachea	17/45 (38%)	5/26 (19%)	12/19 (63%)	0.14 [0.03; 0.51]	0.009
Main bronchi	21/45 (47%)	10/26 (38%)	11/19 (58%)	0.45 [0.13; 1.50]	0.278
Lobar bronchi	20/45 (44%)	10/26 (38%)	10/19 (53%)	0.56 [0.17; 1.85]	0.430
Distal bronchi	23/45 (51%)	12/26 (46%)	11/19 (58%)	0.62 [0.18; 2.04]	0.515
**Extensive involvement**	15/45 (33%)	4/26 (15%)	11/19 (58%)	0.13 [0.03; 0.50]	0.009
**Airwall thickening**					
Trachea	33/45 (73%)	20/26 (77%)	13/19 (68%)	1.54 [0.40; 5.96]	0.597
Bronchi	23/45 (51%)	11/26 (42%)	12/19 (63%)	0.43 [0.12; 1.41]	0.244
Sparing the posterior tracheal membrane	14/45 (31%)	3/26 (12%)	11/19 (58%)	0.09 [0.02; 0.39]	0.003
Irregular	8/45 (18%)	7/26 (27%)	1/19 (5%)	6.63 [1.03; 130.49]	0.090
**Airway stenosis**					
Trachea	14/45 (31%)	8/26 (31%)	6/19 (32%)	0.96 [0.27; 3.56]	0.962
Main/lobar bronchi	17/45 (38%)	7/26 (27%)	10/19 (53%)	0.33 [0.09; 1.13]	0.138
Distal bronchi	15/45 (33%)	9/26 (35%)	6/19 (32%)	1.15 [0.33; 4.20]	0.859
**Tracheal calcifications**	15/45 (33%)	5/26 (19%)	10/19 (53%)	0.21 [0.05; 0.78]	0.045
**Tracheobronchomalacia**	10/20 (50%)	1/5 (20%)	9/15 (60%)	0.17 [0.01; 1.47]	0.148
**Bronchiectasis**	14/45 (31%)	9/26 (35%)	5/19 (26%)	1.48 [0.41; 5.78]	0.619
**Mosaic perfusion**	35/45 (78%)	18/26 (69%)	17/19 (89%)	0.26 [0.04; 1.24]	0.159
**Air trapping**	15/20 (75%)	3/5 (60%)	12/15 (80%)	0.38 [0.04; 3.80]	0.441
**Pulmonary nodule**	7/45 (16%)	6/26 (23%)	1/19 (5%)	5.40 [0.81; 107.21]	0.146
**Pulmonary consolidation**	5/45 (11%)	4/26 (15%)	1/19 (5%)	3.27 [0.44; 67.10]	0.352

Qualitative data are presented as proportion with percentage in parentheses, and quantitative data are presented as median with interquartile range in brackets

*GPA* granulomatosis with polyangiitis, *RP* relapsing polychondritis

^a^The subglottic trachea was out of the scanning field in 7 patients

Indirect CT signs of airway obstruction were frequent with 78% (*n*=35/45) of patients showing mosaic perfusion and 75% (*n*=15/20) of patients with available expiratory CT images presenting air trapping.

Parenchymal abnormalities were less frequent, with pulmonary nodules and consolidations found in 16% (*n*=7/45) and 11% (*n*=5/45), respectively.

### Differentiation between GPA and RP

**Fig. 2:**
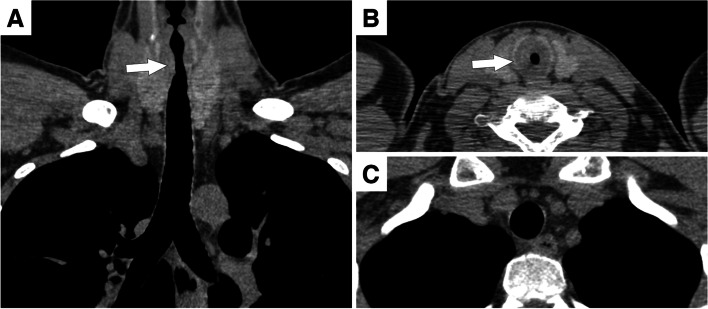
Granulomatosis with polyangiitis. **A** Coronal reformation of the unenhanced chest computed tomography shows focal tracheal wall thickening (arrow). **B** On the axial image, the tracheal wall thickening (arrow) is circumferential and responsible for lumen stenosis. **C** Below, the thoracic trachea is normal

**Fig. 3:**
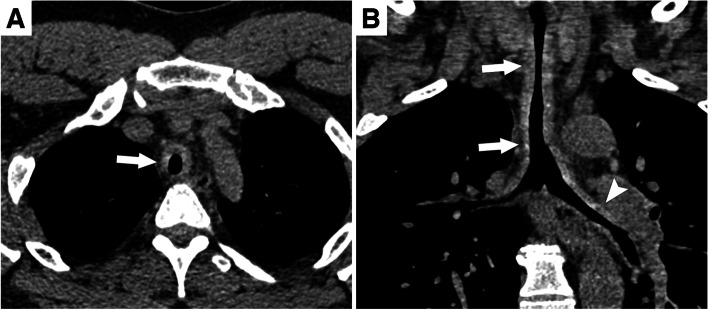
Relapsing polychondritis. **A** Unenhanced chest computed tomography shows tracheal wall thickening sparing the posterior membrane and responsible for lumen stenosis (arrow). **B** On coronal reformation, the tracheal wall thickening is extensive (arrows) and extends to the left main bronchus (arrowhead)

Typical examples of CT findings in GPA and RP patients are illustrated in Figs. 2 and 3. The two observers had an accuracy of 87% (*n*=39/45) and 76% (*n*=34/45), respectively, in differentiating between GPA and RP, based on their experience and subjective assessment. Excluding the cases they classified as indeterminate, their accuracy increased to 91% (*n*=39/43) and 89% (*n*=34/38) with a true positive value for diagnosing GPA of 96% and 92% (*n*=22/23 and *n*=24/26) and a true positive value for diagnosing RP of 85% and 83% (*n*=17/20 and *n*=10/12). Considering indeterminate cases as false negatives and false positives, they then had a sensitivity of 85% (*n*=22/26) and 92% (*n*=24/26) and a specificity of 89% (*n*=17/19) and 53% (*n*=10/29) to diagnose GPA. Their sensitivity for diagnosing RP was 89% (17/19) and 53% (*n*=10/19) while their specificity was 85% (*n*=22/26) and 92% (*n*=24/26).

**Fig. 4:**
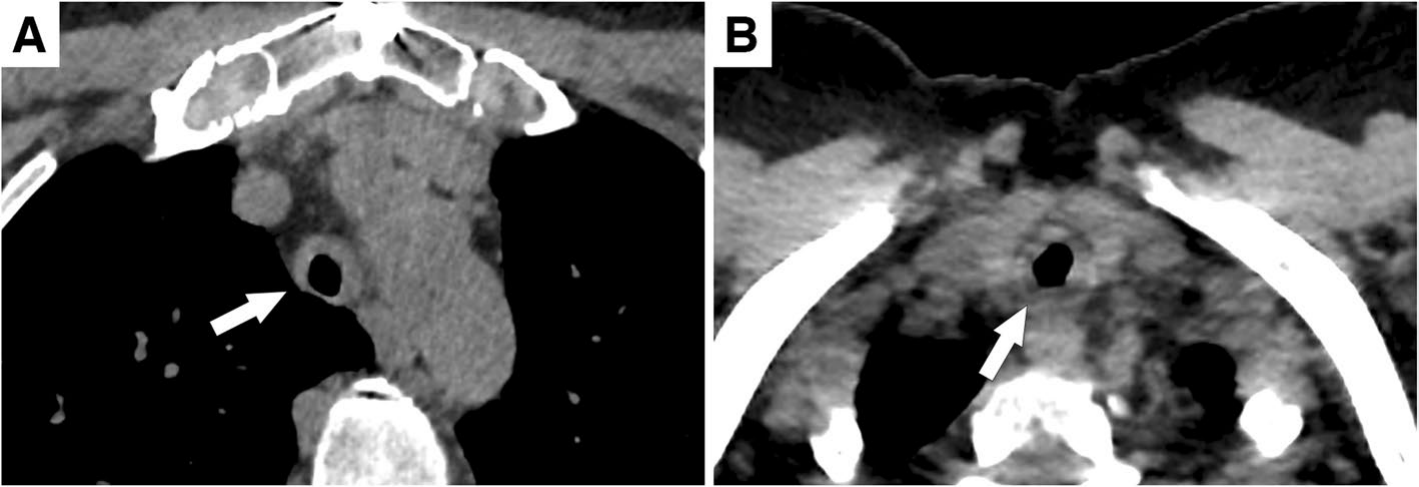
Atypical tracheal wall thickenings. **A** Unenhanced chest computed tomography (CT) shows tracheal wall thickening sparing the posterior membrane (arrow) in a patient with granulomatosis with polyangiitis. **B** Unenhanced chest CT showing circumferential tracheal wall thickening (arrow) in a patient with relapsing polychondritis

Regarding lesion location, univariate analysis showed that involvement of the subglottic trachea was significantly more frequent in patients with GPA (78%, *n*=18/23) than in patients with RP (20%, *n*=3/15) (odds ratio for GPA = 14.40 [95% confidence interval (95% CI): 3.22; 85.07]; *P=0.001*). Of note, the subglottic trachea was out of the scanning field in 7 patients (3 with GPA and 4 with RP). Conversely, involvement of the thoracic trachea was significantly more frequent in patients with RP (63%, *n*=12/19) than in patients with GPA (19%, *n*=5/26) (odds ratio for GPA = 0.14 [95% CI: 0.03; 0.51]; *P=0.009*). Similarly, tracheal involvement sparing of the posterior membrane was more frequently but not exclusively found in patients with RP (58%, *n*=11/19 in RP vs 12%, *n*=3/26 in GPA; odds ratio for GPA = 0.09 [95% CI: 0.02; 0.39]; *P=0.003*). Therefore, tracheal involvement sparing of the posterior membrane can be found in GPA, whereas circumferential involvement can be seen in RP (Fig. 4). Extensive lesions (with a length ≥ 2cm) were also more frequently found in RP (58%, *n*=11/19) than in GPA (15%, *n*=4/26) (odds ratio for GPA = 0.13 [95% CI: 0.03; 0.50]; *P=0.009*) as well as tracheal calcifications (53% [*n*=10/19] in RP vs 19% [*n*=5/26] in GPA; odds ratio for GPA = 0.21 [95% CI: 0.05; 0.78]; *P=0.045*).

Agreement between observers for these five CT findings ranged from moderate for the sparing of the posterior membrane (kappa = 0.55) to almost perfect for extensive involvement (kappa = 0.81) and for involvement of the subglottic trachea (kappa = 0.84) (Table 3). Agreement between observers for the involvement of the thoracic trachea and tracheal calcification was substantial (kappa = 0.76 and 0.75, respectively).

**Table 3:** Agreement between observers (Cohen’s kappa) for the presence of the evaluated CT findings

	Obs1 vs Obs2	Obs 1 vs consensus	Obs 2 vs consensus
**Airway segment involved**			
Subglottic trachea	0.84	0.95	0.89
Cervical trachea	0.77	0.91	0.86
Thoracic trachea	0.76	0.91	0.85
Main bronchi	0.65	0.87	0.77
Lobar bronchi	0.17	0.70	0.33
Distal bronchi	0.59	0.73	0.87
**Extensive involvement**	0.81	0.81	1.00
**Airwall thickening**			
Trachea	0.59	0.77	0.79
Bronchi	0.78	0.91	0.87
Sparing the posterior tracheal membrane	0.55	0.78	0.78
Irregular	0.21	0.21	1.00
**Airway stenosis**			
Trachea	0.50	0.89	0.63
Main/lobar bronchi	0.63	0.77	0.85
Distal bronchi	0.58	0.72	0.85
**Tracheal calcifications**	0.75	1.00	0.75
**Tracheobronchomalacia**	0.20	0.60	0.50
**Bronchiectasis**	0.34	0.60	0.65
**Mosaic perfusion**	0.49	0.93	0.58
**Air trapping**	0.00	0.86	0.00
**Pulmonary nodule**	0.67	0.85	0.81
**Pulmonary consolidation**	0.28	0.51	0.63

*Obs* observer

In addition, patients with GPA tended more frequently to have irregular airway wall thickening (27% [*n*=7/26] vs 5% [*n*=1/19]; odds ratio for GPA = 6.63 [95% CI: 1.03; 130.49]); however, this difference did not reach significance (*P=0.090*).

Multivariate analysis showed that involvement of the subglottic trachea (odds ratio for GPA = 28.56 [95% CI: 3.17; 847.63] *P=0.001*) and extensive involvement (odds ratio for GPA = 0.02 [95% CI: 0.00; 0.43]; *P=0.008*) were the only two independent CT findings that differentiated GPA from RP. Only 6 patients (13%, *n*=6/45) had both subglottic trachea and extensive involvement. Among this patient subset, four had GPA (66%, *n*=4/6), and two had RP (33%, *n*=2/6).

## Discussion

To the best of our knowledge, this study is the first to have evaluated chest CT, blinded to diagnosis, in the differentiation of tracheobronchial involvement in patients with GPA or RP. We found that CT was able to differentiate between these two diseases with an accuracy of 76 to 87% depending on the radiologist and that there were two independent criteria for the differential diagnosis: the presence of subglottic involvement, in favor of GPA, and extensive airway involvement, in favor of RP.

Although many authors consider posterior tracheal membrane sparing as the primary sign to differentiate tracheal involvement in RP from that of GPA [19, 20], our study provides a much more contrasting answer. Indeed, we found that posterior membrane sparing was a criterion in favor of RP in the univariate but not in the multivariate analysis. Additionally, interobserver agreement was only moderate for this criterion, with no observer being more discordant than the other with respect to the consensus. In our study population, 58% of RP patients had tracheal thickening sparing the posterior membrane. Considering only the subset of RP patients with tracheal thickening, the proportion of patients with posterior membrane sparing was 85% (*n*=11/13), which means that 15% of tracheal thickening in RP affected the posterior membrane according to the consensus of radiologists. This is different from the systematic sparing of the posterior membrane often reported in the literature [14, 15, 21]. However, in our study, the radiologists were blinded to the final diagnosis, whereas in several other studies, only patients with a known diagnosis of RP were evaluated, which might have influenced their evaluation. In a recent multicenter study, Catano et al. found circumferential tracheal stenosis in 24% of patients with RP [22]. A posterior tracheal membrane sparing was also found in 12% of our study population with GPA, representing 15% (*n*=3/20) of GPA patients with tracheal thickening. Therefore, our results show that this criterion is complicated to analyze as it does not have a perfect positive or negative predictive value and therefore should be considered accordingly.

Subglottic involvement and the presence of extensive lesions were more robust decision criteria because they showed almost perfect interobserver agreement (kappa ≥0.81). In our series, the subglottic region was the most frequently involved airway segment in GPA patients, affecting 78% of patients. This is also the case in the literature, with subglottic involvement reported in 16 to 23% of GPA patients [23]. However, subglottic involvement is also found in RP patients and was observed in 20% of RP patients in our series. The presence of subglottic stenosis has also been reported in 25% of RP patients in a previous study [15]. In our series, extensive airway involvement was a criterion in favor of RP and was found in 58% of RP patients versus 15% of GPA patients. This is also consistent with the literature as Behar et al. reported diffuse parietal thickening in 66% of RP patients [14]. Catano et al., who recently studied tracheal stenoses in a multicenter cohort of patients with systemic inflammatory diseases, showed that tracheal stenoses in GPA had a median length of 1.5 cm compared with 4 cm in RP [22]. In our study, tracheobronchial lesions were considered diffuse when they were at least 2 cm long.

Interestingly, tracheal calcifications, sometimes reported as a sign suggestive of RP, were also significantly more frequent in RP (53%) than in GPA (19%) but only on the univariate analysis. The frequency of tracheal calcifications in RP was in the range of 10 to 62% reported in other series [15, 21, 22, 24].

This study has several limitations. First, this was a retrospective study and due to the retrospective design, CT evaluation of the cervical region was not available in 7/45 patients. However, this proportion was close in patients with GPA (3/26; 12%) and RP (4/19; 21%). Missing data did not prevent the criterion of subglottic involvement from being retained as an independent criterion in favor of GPA. Similarly, expiratory images were missing in 25/45 patients. Prospective studies in rare diseases are very difficult to perform because of their low incidence. Second, our study was monocentric. However, as our center is a national referral center, receiving patients from many institutions and medical specialties/with a varied distribution of GPA presentations and outcomes, we think that our data likely represents and reflects the entire GPA spectrum and therefore our findings should be applicable to other GPA populations. Finally, the lack of diagnostic criteria for GPA and RP is a limitation. However, patients in our cohort were classified during multidisciplinary meetings at a national reference center, using clinical, biological, radiological, and follow-up information.

## Conclusions

In case of tracheobronchial involvement, it is sometimes difficult to differentiate GPA from RP at initial evaluation. In this study, we have shown that the presence of subglottic involvement and diffuse airway involvement are the two most relevant criteria for the differentiation between GPA and RP on chest CT. We also found that although generally considered a highly suggestive sign, posterior tracheal membrane sparing is nonspecific and an overly subjective sign. These findings can help in differentiating the two diseases that share several other symptoms but require different treatments.

## Data Availability

All data generated or analyzed during this study are included in this published article.

## Abbreviations

CT: Computed tomography
GPA: Granulomatosis with polyangiitis
RP: Relapsing polychondritis
MPO: Myeloperoxidase
ANCA: Anti-neutrophil cytoplasm antibodies
PR3: Proteinase 3
